# Main Bronchus Stenosis Due to Tuberculosis and Ogilvie’s Syndrome: A Case Report of Two Unusual Diseases in the Same Patient

**DOI:** 10.7759/cureus.20420

**Published:** 2021-12-14

**Authors:** Camilo Levi Acuña Pinzon, Jefferson Fabian Nieves Condoy, Roland Kevin Cethorth Fonseca, Claudia Ortiz-Ledesma, Salvador Narváez Fernández

**Affiliations:** 1 Surgery, Hospital Regional de Alta Especialidad del Bajío, León, MEX; 2 General Surgery, Hospital Regional de Alta Especialidad del Bajío, León, MEX; 3 Internal Medicine, Hospital Regional de Alta Especialidad del Bajío, León, MEX; 4 Thorax Surgery, Hospital Regional de Alta Especialidad del Bajío, León, MEX

**Keywords:** mycobacterium tuberculosis, main bronchus stenosis, ogilvie´s syndrome, pulmonar tuberculosis, tracheobronchial tuberculosis

## Abstract

Main bronchus stenosis as a sequel of pulmonary tuberculosis is infrequent and should raise suspicion of other presentations of the infection. Given its non-specific symptomatology and the absence of a specific diagnostic method, tracheobronchial tuberculosis is usually not suspected and diagnosed despite its great impact on quality of life due to the high incidence of stenosis as a consequence. Ogilvie's syndrome, an uncommon condition, requires careful management and surveillance given the risk of ischemia and colonic perforation intrinsic to the disease. We present a case of a patient with main bronchus stenosis secondary to tuberculosis infection and Ogilvie's syndrome post-surgery.

## Introduction

*Mycobacterium tuberculosis* is an aerobic, non-spore-forming bacterium, and the etiological cause of tuberculosis, with the lungs being the most common localization of the disease [1]. Bronchial stenosis is infrequent in pulmonary tuberculosis and usually affects small bronchi, and the involvement of the main bronchus is usually associated with tracheobronchial tuberculosis, a poorly recognized and underdiagnosed entity [2]. On the other hand, Ogilvie's syndrome or acute colonic pseudo-obstruction syndrome is an infrequent entity of unclear physiopathology or etiology [3].

We present a case of a patient with stenosis of the main bronchus secondary to tracheobronchial tuberculosis, with secondary pulmonary fibrosis that required pneumonectomy, who presented Ogilvie syndrome in her postoperative period.

## Case presentation

A 36-year-old woman with no relevant history, a story of 13 months of cough with hyaline expectoration, night sweats, and weight loss of approximately 6 kilograms in three months consulted her primary care hospital where she underwent sputum smear microscopy with positive results for *Mycobacterium tuberculosis*.

She received six months of rifampicin, isoniazid, pyrazinamide, and ethambutol with subsequent negative smears and absence of symptoms. One month after finishing therapy, she presented with anosmia and dysgeusia (no gastrointestinal or other symptoms) and was diagnosed with coronavirus disease 2019 (COVID-19) by polymerase chain reaction (PCR) test receiving symptomatic treatment on an outpatient basis with complete resolution of symptoms.

**Figure 1:**
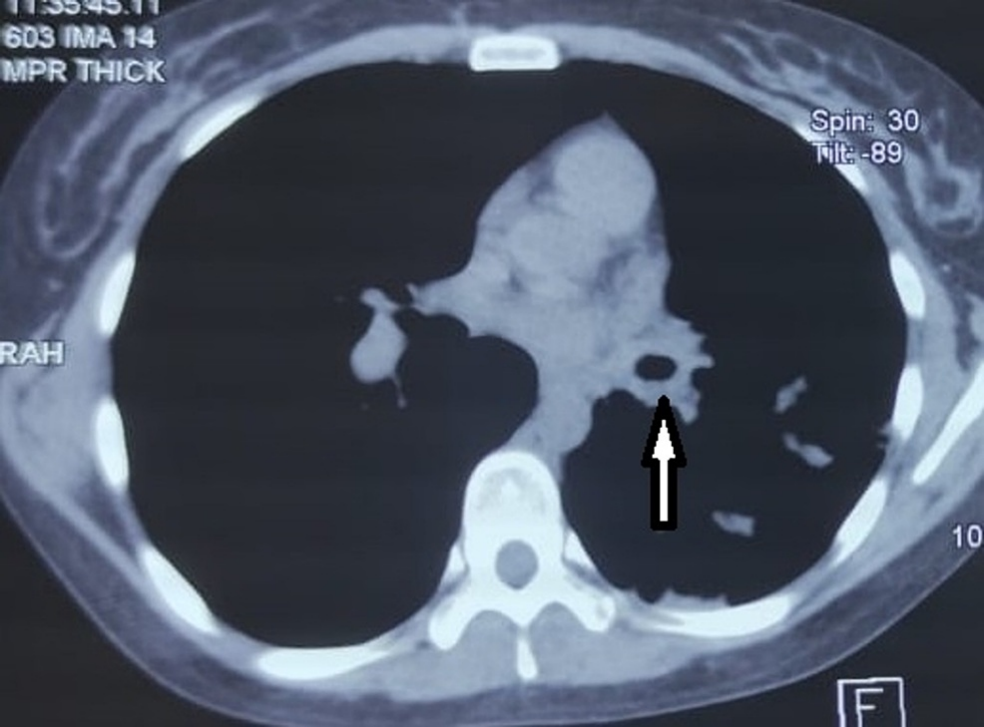
Chest tomography (axial view) showing peribronchial thickening of the left main bronchus (white arrow).

**Figure 2:**
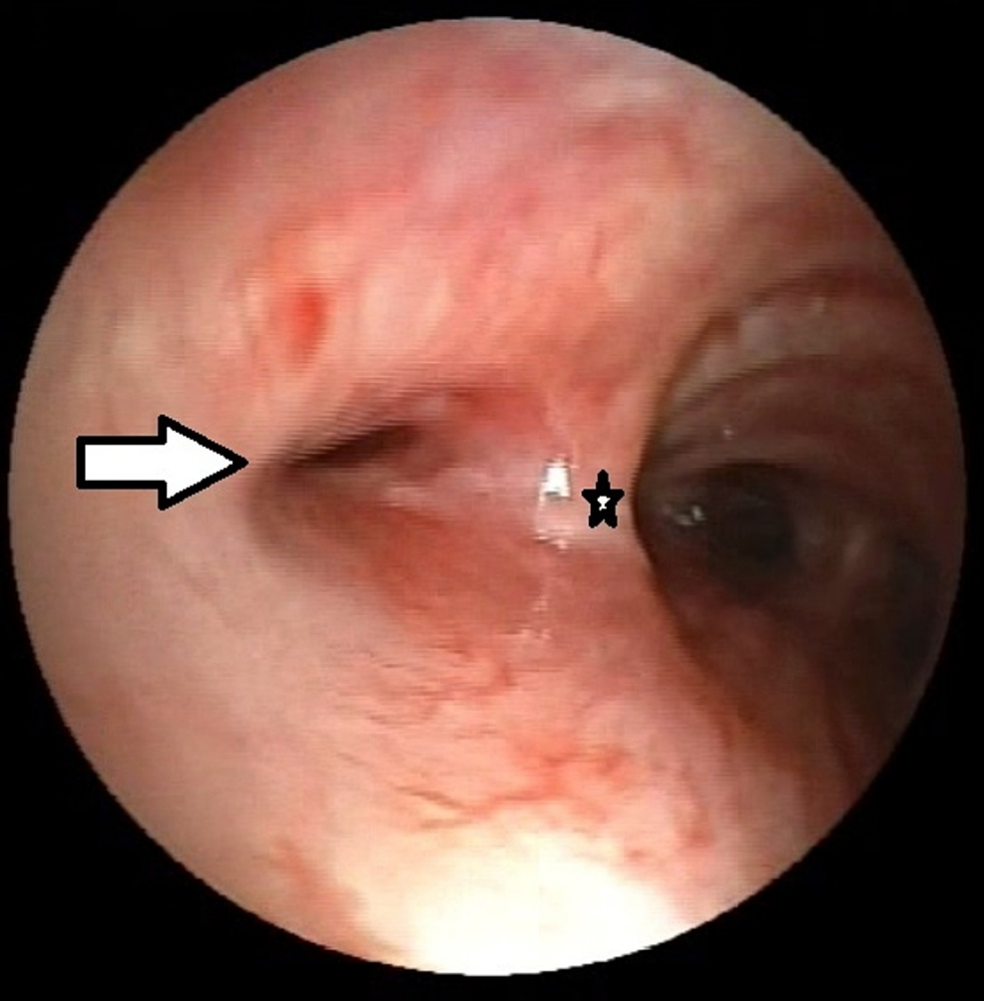
Bronchoscopy showing carina (star) and left main bronchus stenosis (white arrow).

**Figure 3:**
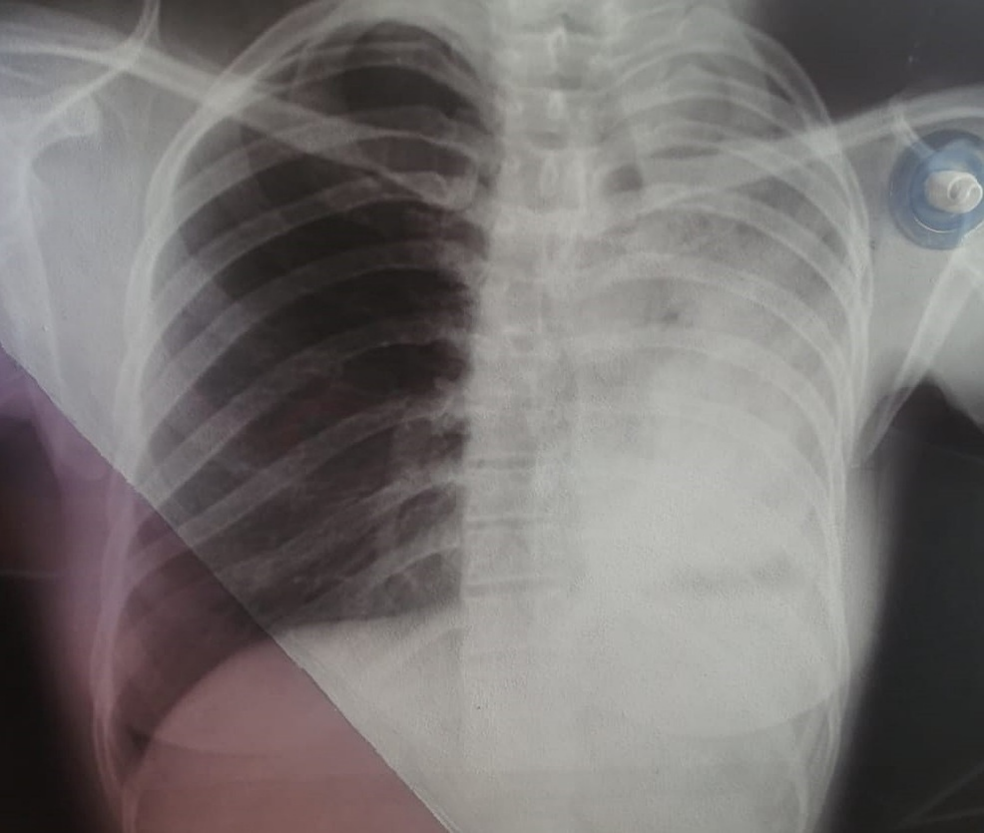
Chest X-ray showing complete fibrosis of the left lung.

**Figure 4:**
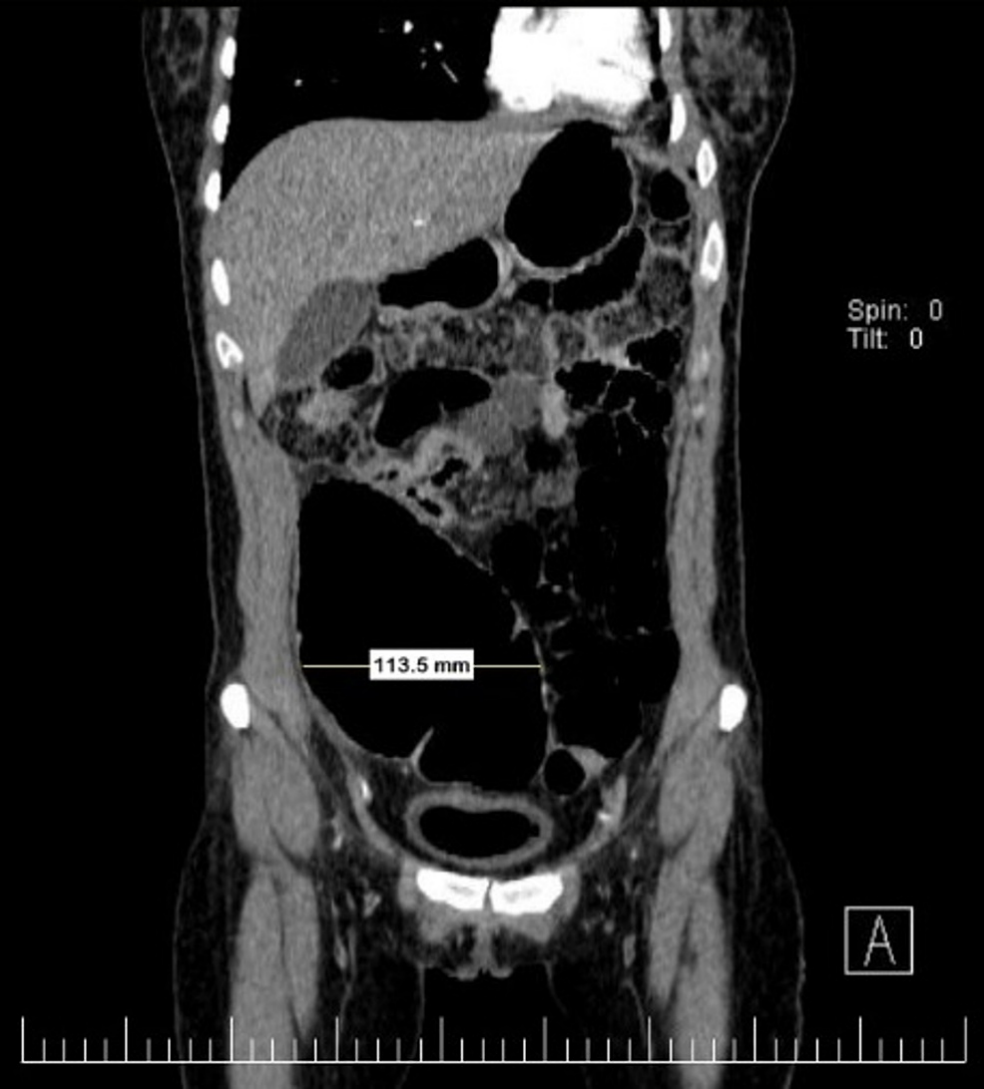
Tomography of the abdomen (coronal view). Dilation of the cecum and ascending colon is observed with a maximum diameter of 113.5 mm as shown in the image.

Two months later, she presented with cough, progressive dyspnea, and decreased functional capacity. A chest tomography was performed, which showed peribronchial thickening of the left main bronchus (Figure 1). A bronchoscopy was performed without being able to cannulate and dilate the main left bronchus (Figure 2). During follow-up chest X-ray and chest tomography, signs of progressive left pulmonary fibrosis were observed (Figure 3) with torpid clinical course, so she underwent pneumonectomy through posterolateral thoracotomy without complications during the procedure. Intraoperative findings revealed pulmonary parenchyma without adhesions, although only around 20% of the parenchyma was correctly aerated; no complications were reported during the procedure. The patient received analgesic management with opioids (buprenorphine) in her immediate postoperative period. On the fourth postoperative day, the patient presented abdominal pain and constipation. Physical examination revealed abdominal distension, decreased frequency of peristaltic sounds, and pain upon palpation of hypogastrium and right iliac fossa. An abdominal CT scan was performed revealing dilatation of the large bowel, primarily in the cecum and ascending colon where the largest diameter was measured as 113.5 mm; no free fluid or other abnormal findings were reported or observed (Figure 4).

Non-surgical management with fasting, opioid suspension, and placement of a rectal tube was performed, presenting resolution of symptoms two days after starting the treatment, so the rectal tube was removed and oral feeding was initiated. Seven days after pneumonectomy, the patient was discharged.

Final histopathological report of surgical specimen informed diffused foci of organized pneumonia with chronic granulomatous inflammation associated with caseous granulomas and microabscesses. Eleven lymph nodes with anthracotic changes and chronic granulomatous inflammation were identified.

On the one-year follow-up after surgery, the patient showed resolution of symptoms, good postoperative recovery, and adequate transition to everyday activities.

## Discussion

The genus Mycobacterium has existed on Earth for 150 million years. The first evidence of tuberculosis in humans dates from 500,000 years ago and its treatment was not possible until 1940. In 1970, it was believed that tuberculosis was going to be eradicated, but the emergence of the human immunodeficiency virus pandemic in 1980 led to the resurgence of tuberculosis worldwide [1].

*Mycobacterium tuberculosis* is shed in droplet nuclei from the airway ciliated mucosa of infected patients. Some of these droplets reach the alveoli of a healthy person, where the bacterium binds to the cell surface of macrophages via complement receptors, mannose receptor, or type A scavenger receptor and subsequently internalizes into the macrophage by phagocytosis. In the phagosome, it inhibits the phagosome-lysosome fusion and multiplies until it ruptures the macrophage, releasing bacilli that are captured by new macrophages perpetuating the process. During primary infection, hematogenous and lymphatic dissemination predominates, reaching several organs and resulting in extrapulmonary tuberculosis. Although it can affect any organ or anatomical part, it is distributed as follows: lymph nodes (47%), pleural cavity (30%), abdomen (10%), bones and joints (8%), central nervous system (82%), and others (3%) [1].

The diagnosis of tuberculosis requires demonstrating the presence of the bacillus by microbiological, cytopathological, or histopathological methods. The classical approach involves phenotypic characterization of colony growth in Lowenstein-Jensen culture medium [1].

In multisensitive tuberculosis and for all new cases of tuberculosis, eight weeks of intensive treatment with isoniazid, rifampicin, pyrazinamide, and ethambutol followed by 16 weeks of isoniazid, rifampicin, and ethambutol is recommended [1,4], the scheme that our also patient received.

The objectives of antituberculosis therapy are to reduce the bacilliferous population rapidly, eradicate persistent bacilli, and prevent antibiotic resistance [4].

Pulmonary resection, despite being the shortest way to remove the infected area, has fallen into oblivion as a treatment for tuberculosis due to the success of antituberculosis drug therapy. Currently, the following are considered as surgical management indications: profuse pulmonary hemorrhage, tension pneumothorax, irreversible progression of the infection, recurrent hemoptysis, cavitation with *Mycobacterium tuberculosis* secretion confirmed after four to six months of antituberculosis therapy, and multidrug-resistant (MDR)/extensively drug-resistant (XDR) infection with therapeutic failure [5].

Among all pulmonary resections for tuberculosis, pneumonectomy constitutes 12% to 15% and is indicated in total destruction of the pulmonary parenchyma or the irreversible lesion of multiple pulmonary segments [5].

Among the main complications of tuberculosis are pneumothorax with or without dissemination of the bacillus in the pleural cavity, massive hemorrhage, bronchiectasis, and infection by Aspergillus in the damaged lung areas. The minor endobronchial disease is common in tuberculosis, but usually involves distal bronchi producing ulceration or stenosis; significant stenosis can occur in larger bronchi, which is very rare [6].

After the resolution of tuberculosis symptoms and negative cultures, our patient suffered from COVID-19. However, this viral disease presents radiological findings such as ground-glass opacities associated with bronchial dilatations and subpleural distortions [7], and bronchial stenosis has not been described as a sequel, so it is unlikely that pulmonary tuberculosis or COVID 19 are the cause of stenosis of the left main bronchus.

Tracheobronchial tuberculosis is defined as a tuberculous infection involving the tracheobronchial tree; it may be secondary to direct infiltration from the lungs, implantation of microorganisms by secretions or sputum, hematogenous dissemination, lymphatic dissemination, or erosion of lymph nodes within the trachea or bronchi. It appears frequently in women with ages ranging between the second and third decade of life. The bronchial lumen diameter is significantly narrower in women than in men, so that retained sputum may make bronchi vulnerable to mycobacterium infection, explaining the higher frequency of the disease in women [2].

The potential for developing bronchial stenosis is high and can be predicted from the bronchoscopy appearance of the mucosa as demonstrated by and Chung et al. in his comprehensive study. Stenosis usually develops from bronchial ulcers that evolve through three stages: active (Stage A), healing (Stage H), and scarring (Stage H), the latter being the most important in pathogenesis. As part of the treatment, in addition to antituberculous therapy, the addition of a steroid (prednisone) is recommended [8].

Returning to the case of our patient; although it was not diagnosed before the stenosis was symptomatic, we consider it highly probable that stenosis was secondary to tracheobronchial tuberculosis rather than pulmonary tuberculosis, given the infrequency of major bronchial disease in the latter and the epidemiology of the former. As mentioned previously, extrapulmonary tuberculosis most commonly presents in the central nervous system, lymph nodes, and pleural cavity. Tracheobronchial tuberculosis is part of other sites of dissemination that together represent 3% [1]; however, another bibliography describes tracheobronchial tuberculosis in 10%-50% of patients with active pulmonary tuberculosis [6], a percentage that does not correlate with what was found in clinical practice. Despite being infrequent, tracheobronchial tuberculosis will develop stenosis even despite adequate treatment in more than 90% of cases [6], and in 68% during the first six months [8].

A wide range of modalities has been described to restore airway patency. Balloon dilatation, stents, laser photo-resection, argon plasma coagulation, and cryotherapy are some of the treatments usually used when the stenosis is mild or moderate. For severe or refractory stenosis, surgical treatment should be chosen. The choice of surgical treatment should be taken on an individualized approach according to the extent of the stenosis and the sequels on the pulmonary parenchyma; it can vary from surgical bronchoplasty to lobectomy or pneumonectomy [8,9].

When the bronchoscopic attempt to permeabilize the patient's airway failed and taking the fibrotic process on the pulmonary parenchyma into account, the only option to ensure the effective treatment was surgical. In this case, pneumonectomy was chosen due to main bronchus involvement. Posterior to the surgical event, our patient developed abdominal pain, and tomographic findings of the cecum and ascending colon dilation related to intestinal obstruction or pseudo-obstruction were found.

Colonic pseudo-obstruction, also known as Ogilvie's syndrome, is a form of colonic dilation that occurs in the absence of an underlying mechanical or anatomic cause. Its incidence has been estimated as approximately 100 cases per 100,000 hospitalizations each year and primarily affects elderly individuals with multiple co-morbidities, hydric and electrolyte disturbances, polypharmacy, and poor functional status with an average onset age around the sixth decade of life. It has also been reported in healthy individuals after traumatic injury or surgery [3].

Classically, this dilation is confined to the cecum and ascending colon; it usually manifests three to five days after surgical trauma; however, the development of this pathology is unpredictable. The cecal diameter correlates directly with the risk of complications such as ischemia, peritonitis, or perforation with an especially increased risk after the colon reaches a diameter equal to or greater than 12 cm. The duration of the disease is the main predictor for ischemia or perforation; a dilation that persists for more than five to six days confers the highest risk and directly impacts mortality [3].

Its pathophysiology is unclear, and it is believed that there is an injury to the colonic motor system with dysfunction or imbalance of the autonomic nervous system and reduced activity of stimulatory neurotransmitters, mainly acetylcholine [9]. Inflammatory cytokines released by surgical trauma that stimulate inhibitory neurotransmitters and nitric oxide associated with vagal activation is another theory for the development of the syndrome [10].

The main goal of treatment is intestinal decompression. Options include conservative therapy (observation, fasting, rectal, or nasogastric tube), interventional management (pharmacologic and endoscopic management), and surgical therapy when there is a failure of conservative treatment or ischemia/perforation. The conservative approach can be given for up to 72 hours with a success rate of up to 90% after which pharmacological therapy should be initiated. The main drug used is neostigmine at a dose of 2 mg intravenously applied over a period of two to five minutes. Endoscopic management for decompression is considered technically difficult and success rates are directly related to the operator's experience. Resolution of the disease is defined as the passage of flatus or stool and or decreasing cecal diameter [3].

A study comparing conservative management (fasting, nasogastric, or rectal tube placement for decompression and opioid drug withdrawal) and interventional management (neostigmine administration, endoscopic decompression, and surgery) showed high effectiveness with conservative management and no evidence to favor interventional over conservative management [11].

Fortunately, our patient resolved the pseudo-obstruction with conservative management alone with fasting and rectal probe for decompression since correcting electrolyte disturbance was not necessary, given the normal values of these in blood samples.

Ogilvie's syndrome has been described in gynecological procedures [12], associated with COVID 19 infection [13] and post-pneumonectomy [14] among other pathologies and procedures.

The relationship between severe acute respiratory syndrome coronavirus 2 (SARS-CoV-2) and intestinal involvement through angiotensin-converting enzyme type 2 receptor has been widely described in the literature; however, there are few reported cases of COVID-19 infection associated with Ogilvie's syndrome [13,15,16]. All the reported cases of the association of these two pathologies have in common the age greater than or equal to 60 years and the short period of time between the symptoms and the appearance of Ogilvie's syndrome. In our case, the patient presented mild SARS-CoV-2 infection managed symptomatically and in an outpatient setting with complete resolution of symptoms. Months after the infection, she presented with Ogilvie's symptoms in the post-surgical period, for which an association is unlikely.

It is impossible to make a specific association of the syndrome with the pneumonectomy performed or the previous infection by COVID-19 but we believe that both events were related to its appearance.

## Conclusions

Main bronchus stenosis is an uncommon sequel of pulmonary tuberculosis, and tracheobronchial tuberculosis should be suspected, a pathology that is usually overlooked and underdiagnosed but has a great impact on the quality of life of affected patients.

Ogilvie's syndrome is relatively uncommon in young people but its proper management is crucial, given the risk of ischemia and colonic perforation intrinsic to the pathology.
